# Supplementary material for: Fecal Bile Acids and Neutral Sterols Are Associated with Latent Microbial Subgroups in the Human Gut
Source: Metabolites. 2022 Sep 8;12(9):846. doi: 10.3390/metabo12090846 (PMC9504437; doi:10.3390/metabo12090846)

Supplementary Figures

Supplementary Figure S1. Heat map displaying the results of a sensitivity analysis excluding participants taking lipid-lowering medications, including statins.

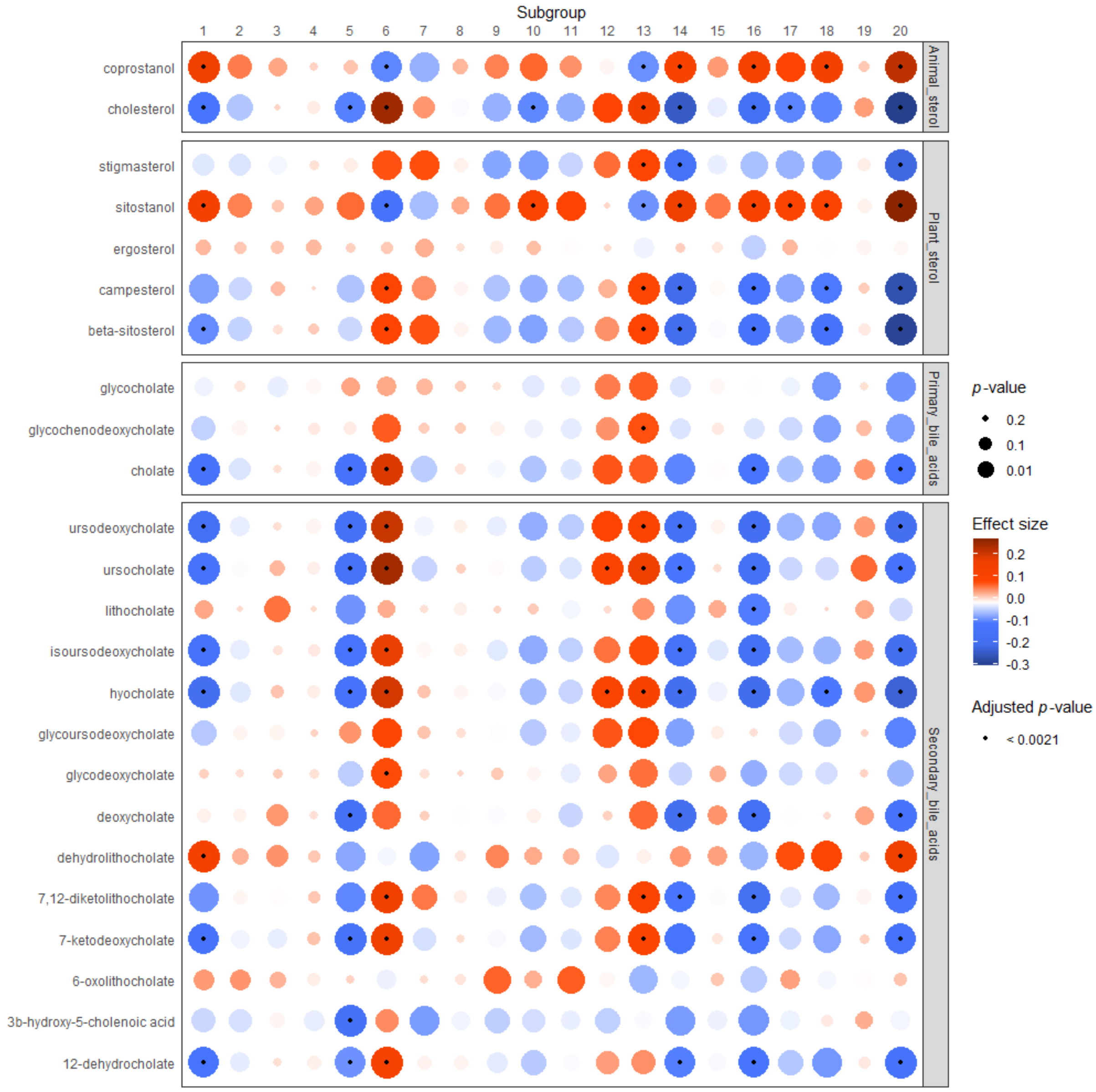

Supplementary Figure S2. Non-linear associations between stigmasterol and the 20 microbial subgroups. The blue line represents the non-linear association while the dotted line represents the linear trend. p1 and p2 represent the p-values for the linear and quadratic terms, respectively, for the non-linear model.

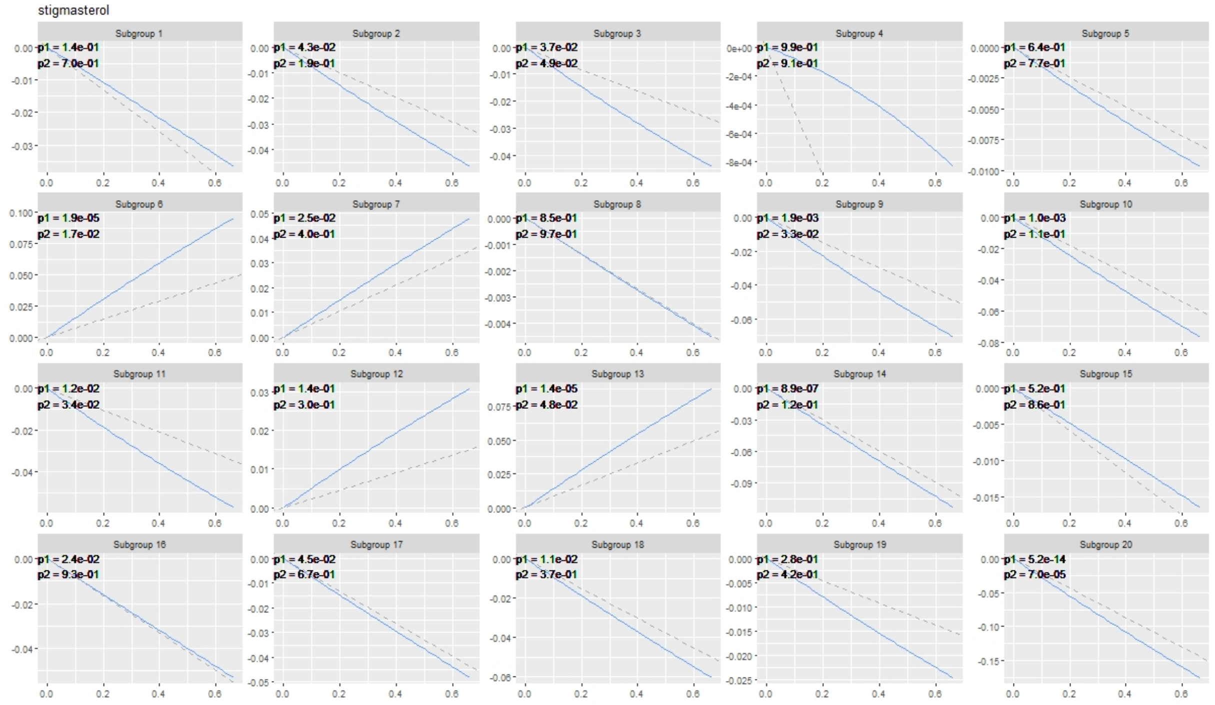

Supplementary Figure S3. Non-linear associations between sitostanol and the 20 microbial subgroups. The blue line represents the non-linear association while the dotted line represents the linear trend. p1 and p2 represent the p-values for the linear and quadratic terms, respectively, for the non-linear model.

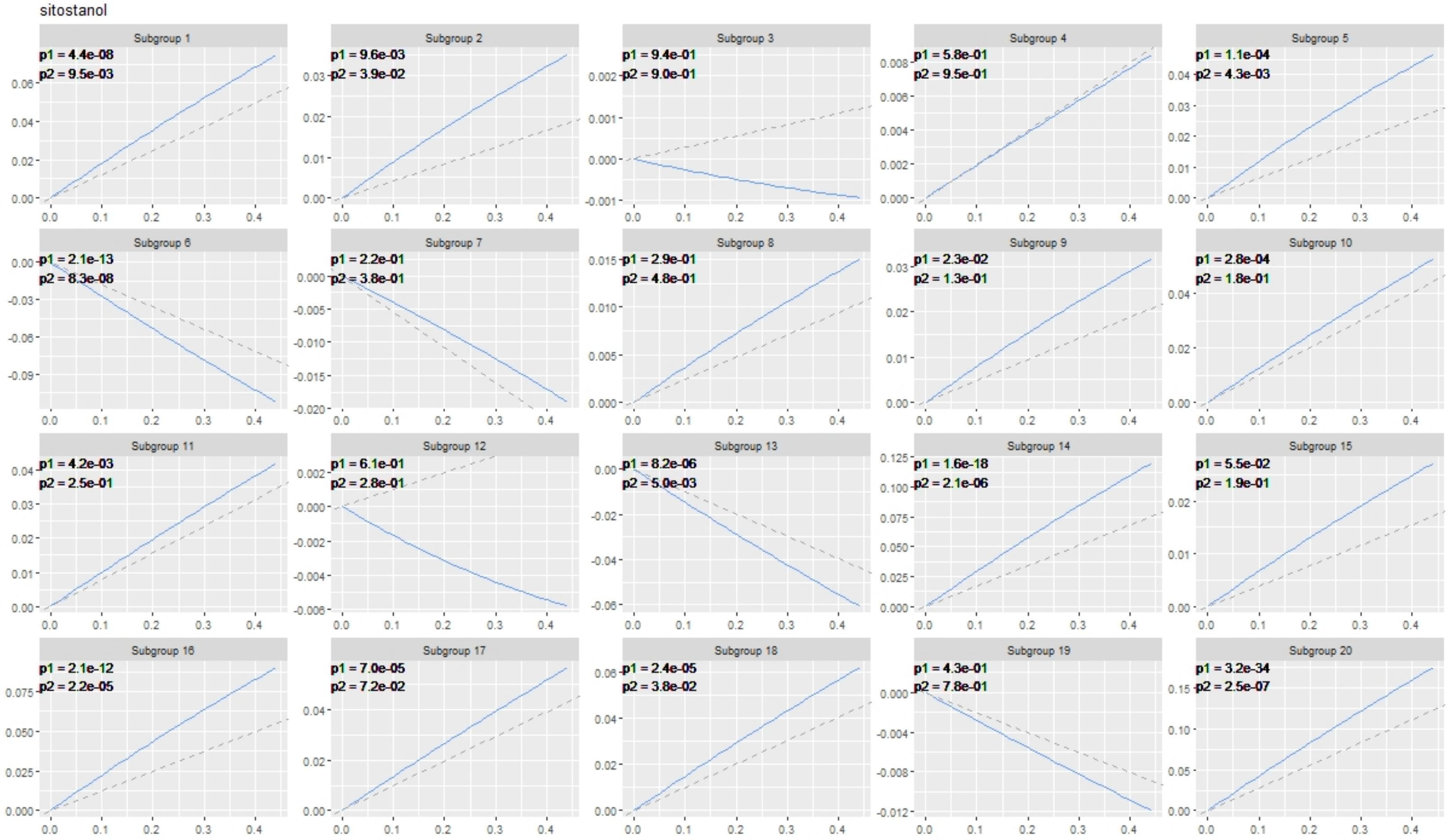

Supplementary Figure S4. Non-linear associations between ergosterol and the 20 microbial subgroups. The blue line represents the non-linear association while the dotted line represents the linear trend. p1 and p2 represent the p-values for the linear and quadratic terms, respectively, for the non-linear model.

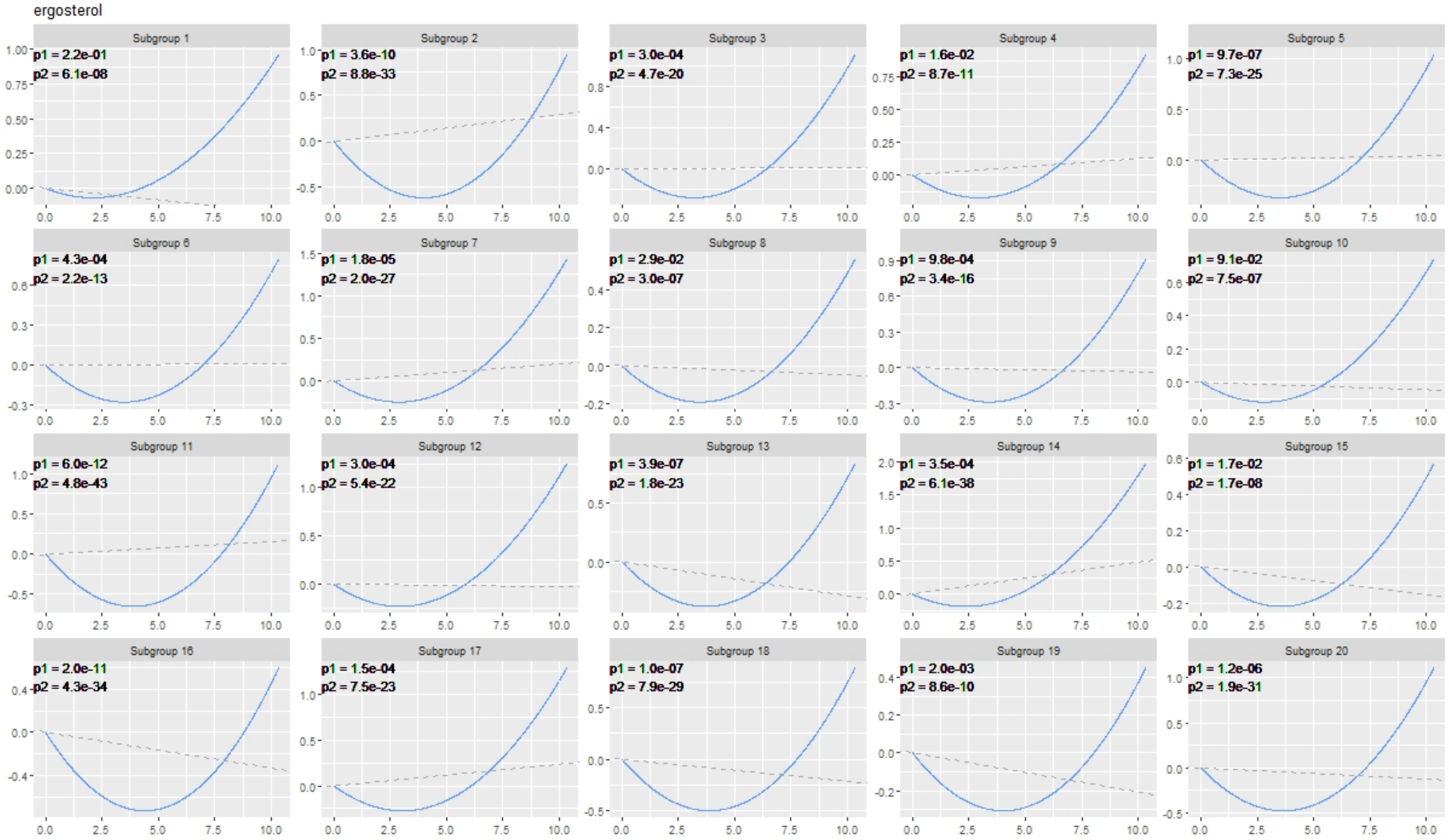

Supplementary Figure S5. Non-linear associations between campesterol and the 20 microbial subgroups. The blue line represents the non-linear association while the dotted line represents the linear trend. p1 and p2 represent the p-values for the linear and quadratic terms, respectively, for the non-linear model.

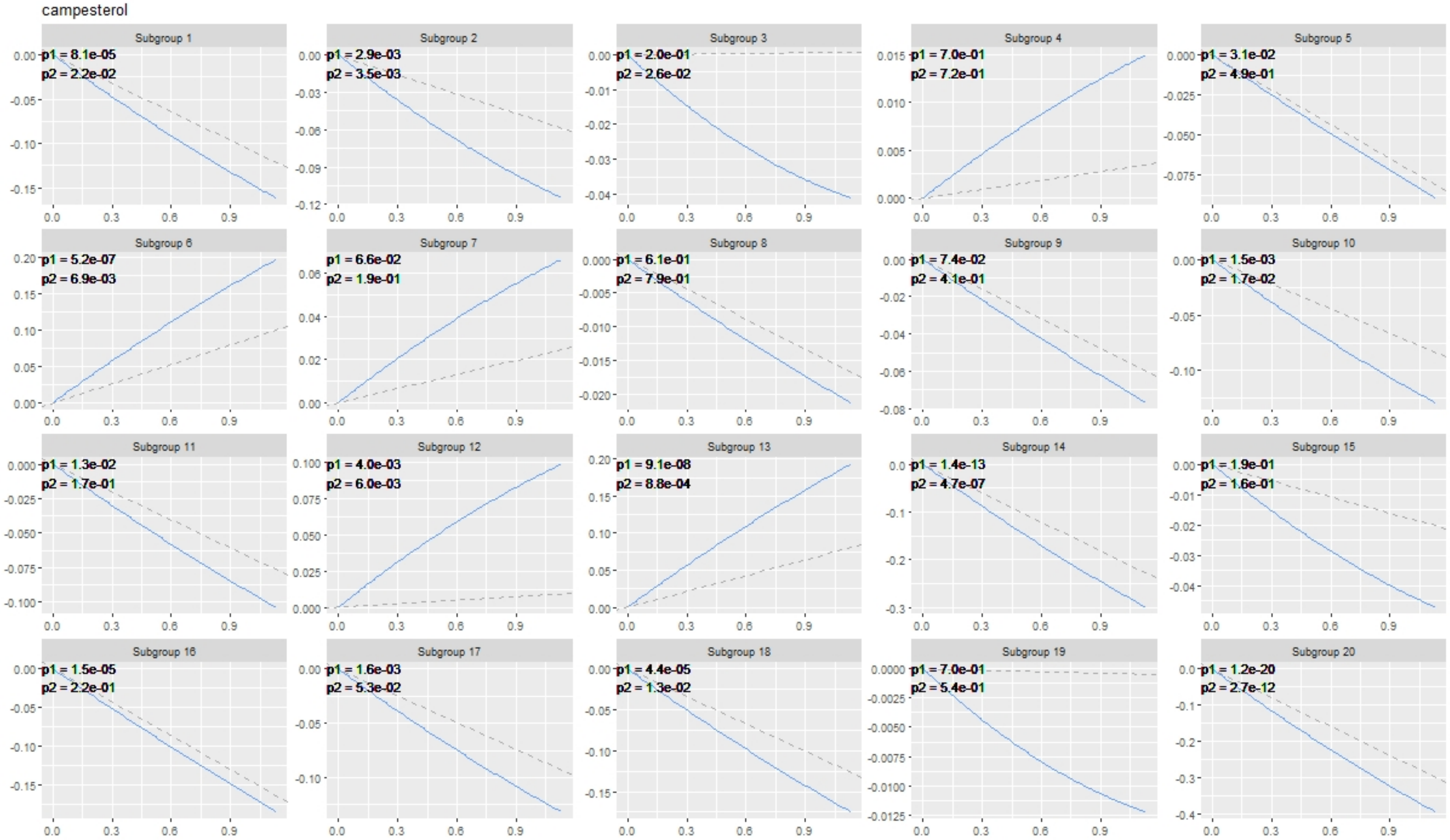

Supplementary Figure S6. Non-linear associations between beta-sitosterol and the 20 microbial subgroups. The blue line represents the non-linear association while the dotted line represents the linear trend. p1 and p2 represent the p-values for the linear and quadratic terms, respectively, for the non-linear model.

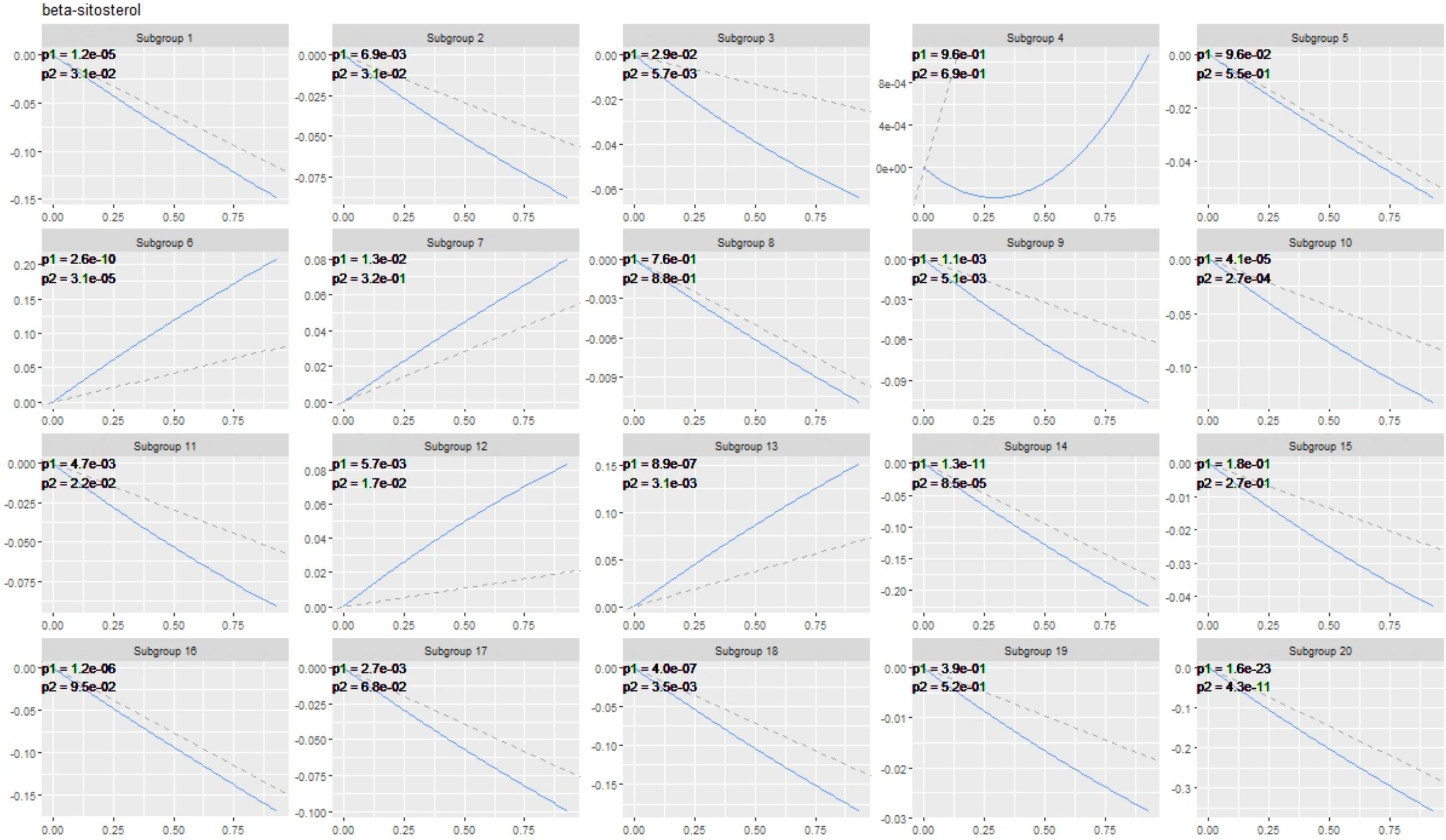

Supplement: Supplementary file 1 [file metabolites-12-00846-s001.zip › Supplementary Figures S1-S6.pdf]
