# Supplementary material for: Fecal Bile Acids and Neutral Sterols Are Associated with Latent Microbial Subgroups in the Human Gut
Source: Metabolites. 2022 Sep 8;12(9):846. doi: 10.3390/metabo12090846 (PMC9504437; doi:10.3390/metabo12090846)
Supplement: Supplementary file 1 [file metabolites-12-00846-s001.zip › Supplementary Table S9.pdf]

Supplementary Table S9. Metabolite synonyms.

**Metabolite names**	**Synonyms**
Stigmasterol	3β-Hydroxy-24-ethyl-5,22-cholestadiene, 5,22-Stigmastadien-3β-ol
Sitostanol	dihydrositosterol; (3β)-stigmastan-3-ol; (3β,5α)-stigmastan-3-ol
Beta-sitosterol	22,23-Dihydrostigmasterol, 5-Stigmasten-3β-ol, α-Dihydrofucosterol, 24α-Ethylcholesterol
Campesterol	24α-Methyl-5-cholesten-3β-ol, 24(R)-Ergost-5-en-3β-ol
Ergosterol	3β-Hydroxy-5,7,22-ergostatriene, Provitamin D_2, 5,7,22-Ergostatrien-3β-ol,
Cholesterol	3β-Hydroxy-5-cholestene, 5-Cholesten-3β-ol,
Coprostanol	5β-Cholestan-3β-ol
	**salt of …**
Cholate	3α,7α,12α-Trihydroxy-5β-cholan-24-oic acid
Glycocholate	N-(3α,7α,12α-Trihydroxy-24-oxocholan-24-yl)glycine
Glycochenodeoxycholate	N-(3α,7α-Dihydroxy-24-oxocholan-24-yl)glycine
12-dehydrocholate	5β-Cholanic acid-3,5,12-trione, (5β)-3,7,12-Trioxocholan-24-oic acid, 3,7,12-Trioxo-5β-cholanic acid
Lithocholate	3α-Hydroxy-5β-cholan-24-oic acid, 3α-Hydroxy-5β-cholanic acid, 5β-Cholan-24-oic acid-3α-ol
6-oxolithocholate	-
7,12-diketolithocholate	-
Dehydrolithocholate	-
3b-hydroxy-5-cholenoic acid	(25R)-cholest-5-en-26-oic acid, 3β-hydroxy
Deoxycholate	3α,12α-Dihydroxy-5β-cholanic acid
Glycodeoxycholate	N-(3α,12α-Dihydroxy-24-oxocholan-24-yl)glycine,
Glycoursodeoxycholate	*N*-(3α,7β-Dihydroxy-5β-cholan-24-oyl)glycine, *N*-[(3α,5β,7β)-3,7-Dihydroxy-24-oxocholan-24-yl]glycine
Ursodeoxycholate	5β-Cholan-24-oic acid-3α,7β-diol, 3α,7β-Dihydroxy-5β-cholan-24-oic acid, 7β-Hydroxylithocholic acid,
Isoursodeoxycholate	-
7-ketodeoxycholate	-
Hyocholate	3α,6α,7α-trihydroxy-5β-cholanic acid
Ursocholate	3α,7ß,12α-trihydroxy-5ß-cholan-24-oic acid
